# Supplementary material for: A reactive neural network framework for water-loaded acidic zeolites
Source: Nat Commun. 2024 May 17;15:4215. doi: 10.1038/s41467-024-48609-2 (PMC11101627; doi:10.1038/s41467-024-48609-2)
Supplement: Supplementary file 1 — Supplementary Information [file 41467_2024_48609_MOESM1_ESM.pdf]

Supplementary Information

A reactive neural network framework for water-loaded acidic zeolites

Andreas Erlebach,[1]* Martin Šípka,[1,2] Indranil Saha,[1]
Petr Nachtigall,[1] Christopher J. Heard,[1] Lukáš Grajciar[1]*

[1]*Department of Physical and Macromolecular Chemistry, Charles University, Hlavova 8, 128 43 Praha 2*

[2]*Mathematical Institute, Faculty of Mathematics and Physics, Charles University, Sokolovská 83, 186 75 Prague, Czech Republic*

1. Supplementary methods

Supplementary Table 1 summarizes the number of T-sites, aluminum atoms (or Brønsted acid sites (BAS)), framework density, database ID for hypothetical zeolites (see Ref. 1), and Si-O ring sizes (calculated using the algorithm of Crum et al.),[2] and the number of water molecules of all generated initial structures used for DFT database generation. These initial structures were selected to get high structural diversity of silica zeolites in terms of atomic density and similarity of atomic environments. We first selected 7 zeolites by Farthest Point Sampling[3,4] together with the SOAP descriptor[5] (SOAP-FPS) from a hypothetical zeolite database which contains more than 330k siliceous zeolites. This procedure allows to generate a subset of structures with maximally distinct atomic environments in the zeolite structure. We also added three existing zeolites with different framework densities (14.9 – 20.5 Si nm^{-3}) and a low-density, two-dimensional silica bilayer (BL) to ensure optimal coverage of the zeolite configuration space (see also Ref. 1 for further details). The selected initial structures contain ring sizes from 3-membered rings to 14-membered rings.

Supplementary Figure 1 shows the framework density as a function of SOAP similarity distance of atomic environments with respect to α-quartz for 330k hypothetical zeolites, the selected training set and two test sets (1000 configurations for single-point calculations, 7 for structures optimization, see Supplementary discussion and Supplementary Table 4). The aluminum substituted zeolites were created with a random Al distribution according to Löwenstein's rule. Three AIMD runs (at 1200, 2400, and 3600 K) were performed for the 10 zeolites that contain Al (three Si/Al ratios) together with the four listed water loadings (10 topologies x 3 Si/Al x 4 water loadings x 3 temperatures = 360 runs). For the purely siliceous frameworks, only water-loaded cases were used for AIMD simulations (10 topologies x 1 Si/Al x 3 water-loadings x 3 temperatures = 90 runs). We also performed AIMD runs for three water-loaded models of a silica bilayer *in vacuo* (12 Å vacuum layer) taken from the silica database generated in our previous work[1] (3 water-loadings x 3 temperatures = 9 runs). Structurally distinct configurations were selected from every AIMD run using SOAP-FPS employing a cutoff r_{cut} = 6 Å, n_{max} = 8 basis functions (gaussian type orbitals) and spherical harmonics up to degree l_{max} = 8.

Supplementary Table 1. Number of T-sites (N_t), aluminum atoms or BAS (N_{Al}) and water molecules (N_w), database index (ID) of the hypothetical zeolite database published in Ref. 1, and framework density (FD in N_t per nm^3) of the initial structures constructed for database creation using existing zeolite topologies (CHA, SOD, MVY), hypothetical frameworks (HYP) and a silica bilayer (BL). Only water-loaded configurations ($N_w > 0$) were used for purely siliceous cases ($N_{Al} = 0$).

Topology	ID	FD	Ring sizes	N_t	N_{Al}				N_w			
CHA	-	14.9	4-6-8-12	12	0	1	3	6	0	2	8	14
SOD	-	17.8	4-6-12	12	0	1	3	6	0	1	4	8
MVY	-	20.5	4-6-10	24	0	2	6	12	0	1	4	7
HYP1	17883	23.2	4-5-6-7-10	14	0	1	4	7	0	1	2	3
HYP2	2251	19.5	4-5-6-8-10-14	16	0	1	3	5	0	1	5	10
HYP3	17089	20.9	4-5-6-7-8-9-10	32	0	1	7	14	0	1	7	12
HYP4	46458	21.5	4-5-6-8-10	28	0	1	5	10	0	1	3	5
HYP5	4552	20.1	4-5-6-7-8-12	32	0	1	5	10	0	1	4	7
HYP6	9330	19.2	3-4-5-6-7-8-10-11-12	16	0	1	3	6	0	1	5	9
HYP7	72920	15.8	4-5-6-8-10-14	24	0	1	5	9	0	2	12	22
BL	-	9.7	4-5-6-10	24	0	-	-	-	-	5	32	59

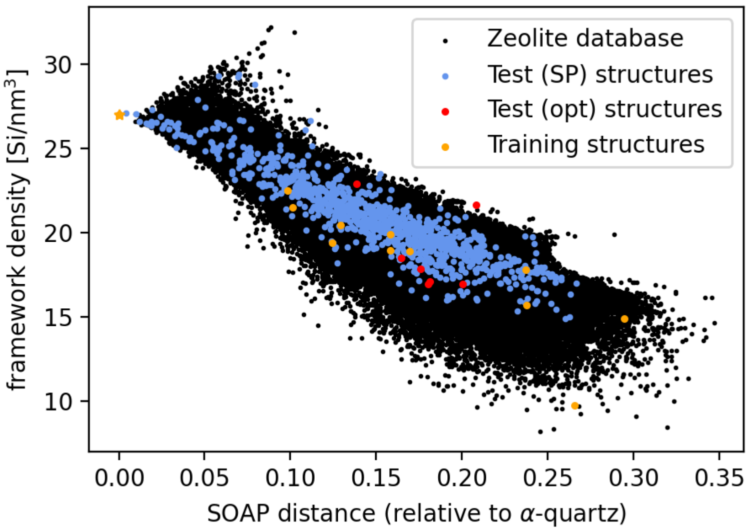

Supplementary Figure 1 Framework density as a function of SOAP distance relative to α-quartz (orange star) for the hypothetical zeolite database (Ref. 1), structures used for NNP training, a large test set using single-point calculations (Test SP), and a smaller test set for structure optimizations (Test (opt)) at the NNP and DFT level (see Supplementary discussion and Supplementary Table 4).

The 153 geometrically optimized initial structures with optimized unit cells $\mathbf{M}_0 = (\mathbf{a}, \mathbf{b}, \mathbf{c})$ and lattice vectors $\mathbf{a}, \mathbf{b}, \mathbf{c}$ were systematically deformed using 70 different (an)isotropic deformations $\mathbf{M}_\mathrm{d} = (\mathbf{I} + \boldsymbol{\varepsilon})\mathbf{M}_0$ defined by the symmetric 3×3 deformation matrix $\boldsymbol{\varepsilon}$. The deformation factor $\varepsilon_{ij} = \pm d$ determines the magnitude of each deformation. Here, we employed three deformation factors $d = 0.015, 0.03, 0.045$ yielding 210 perturbed structures for each initial configuration (see Ref. [1] for further details).

Supplementary Figure 2 depicts three t-distributed stochastic neighbor embedding (t-SNE) plots of the final SCAN+D3(BJ) database and the performed generalization tests (see also Figure 1) which are out-of-domain (OOD) of the training dataset. The t-SNE algorithm maps similar (close lying) points in a high-dimensional space to a lower (here two) dimensional space for data visualization. Here, we used as t-SNE input the SchNet NNP representation vectors averaged over each structure in the generated DFT dataset and the SCAN+D3(BJ) calculated subsets of every generalization test trajectory. Supplementary Figure 1 shows that similar SchNet representations of an atomic configuration correspond to structures similar in energy and chemical composition (Al_2O_3 and H_2O content).

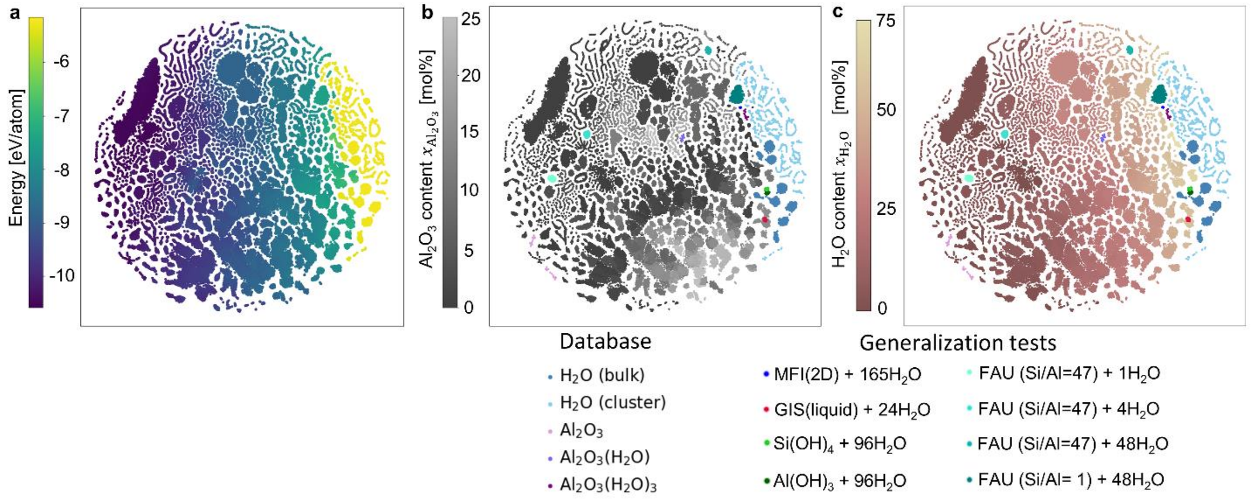

Supplementary Figure 2 t-SNE plots of average representation vectors of the trained SchNet NNP for the database and generalization tests (see Figure 1): **a** atomic energy, **b** Al_2O_3 content and **c** water content as a function of the first two t-SNE components. Water clusters, bulk water, alumina polymorphs (as part of the database) and generalization tests (not part of the database) are highlighted in **b** and **c**.

Supplementary Figure 3 shows a representative learning curve of a training run as well as energy and force RMSE as a function of training set size (different train-validation-test splits) and relevant SchNet hyperparameters. The learning curve shows relatively large fluctuations in the early training stage at high learning rates due to the small batch size. The lowest NNP energy and force errors were found for training set sizes of 173907 (70/10/20 split) and 198800 (80/10/10 split). Smaller training set sizes show only slightly larger errors demonstrating that the data set size is not a limiting factor of the NNP accuracy, especially for even more data-efficient, equivariant NNP architectures (e.g., PaiNN).[6] In addition, the NNPs show best performance with the SchNet hyperparameters used in this work (6 interaction blocks, cutoff radius 6 Å) in line with previous studies.[7,8] The energy and force MAEs and RMSEs of the trained NNP ensemble are summarized in Supplementary Table 2.

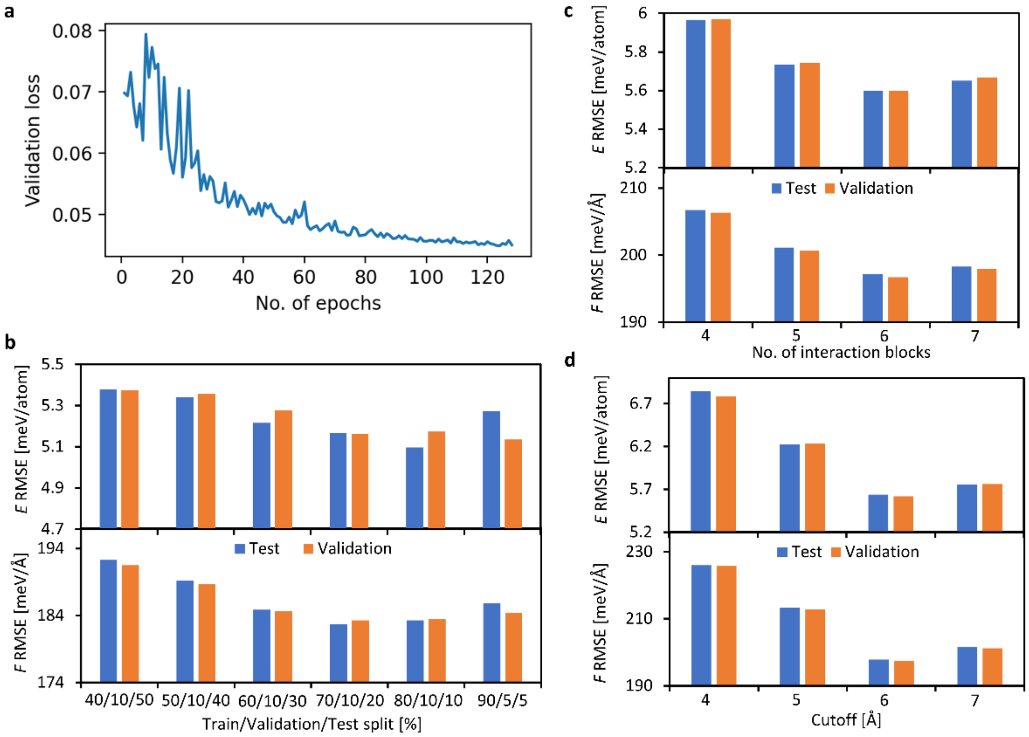

Supplementary Figure 3 Learning curve for typical training run (a) as well as energy and force RMSE as a function of train-validation-test split (b), number of interaction blocks (c), and neighbor cutoff radius (d). The NNPs trained in this work used six interaction blocks, a 6 Å cutoff and the 80/10/10 train/validation/test split.

Supplementary Table 2. Mean absolute (MAE) and root mean square error (RMSE) for the database test sets.

NNP ensemble	Energy [meV atom^{-1}] MAE	Energy [meV atom^{-1}] RMSE	Forces [meV Å^{-1}] MAE	Forces [meV Å^{-1}] RMSE
1	3.4	5.1	118.1	183.3
2	3.4	5.3	119.6	187.8
3	3.6	5.5	120.8	187.7
4	3.4	5.2	118.2	184.2
5	3.5	5.5	121.2	188.3
6	3.4	5.3	119.5	185.3

2. Supplementary discussion

In this work, we use two energy error metrics to test the NNP performance with respect to SCAN+D3(BJ): a reaction energy error ΔE_r (see Eq 1) to compare test systems with different chemical composition and the error $\Delta\Delta E$ of relative energy ΔE with respect to a reference structure with the same chemical composition, e.g., the initial structure of an MD trajectory. Supplementary Figure 4 shows the energy error distributions of both metrics for all generalization (OOD) tests calculated at the NNP and ReaxFF level. Supplementary Table 3 also lists both energy RMSEs,

ΔE_r and $\Delta\Delta E$, force RSMEs and the covariances that quantify potential correlations of the DFT energy and forces.

The error distributions ΔE_r show an offset in most test cases leading to larger RMSEs compared to $\Delta\Delta E$, in particular for ReaxFF. This offset is almost removed for the $\Delta\Delta E$ distributions together with 2-6 times lower RMSEs. As an example, the relative energy RMSE $\Delta\Delta E$ of GIS(T=3000K)+24H_2O (NNP: 6 meV atom^{-1}; ReaxFF: 83 meV atom^{-1}) are about two times lower than ΔE_r. Overall, the NNP accuracy outperforms ReaxFF by about one order of magnitude in both energy metrics and forces. We also calculated the covariances between the NNP errors for total energy ΔE_{tot} and forces ΔF and the SCAN+D3(BJ) calculated energies/forces (Supplementary Figure 6 shows an example for such a dependency). The covariances were calculated using the total energy and forces after subtracting the mean and dividing by the standard deviation of each data set to obtain normalized data. Most importantly, the NNPs show force covariances lower than 0.24 demonstrating random force errors so that the NNPs do not bias MD samplings to incorrect configurations. Also, the NNP energy errors are mostly random and, therefore, the NNPs do not introduce systematic energy errors. Only in case of FAU(Si/Al=1)+48H_2O there is a mild correlation of the NNP energy errors with a covariance of 0.59. However, these errors are on average below 1 meV/atom which is within DFT accuracy.

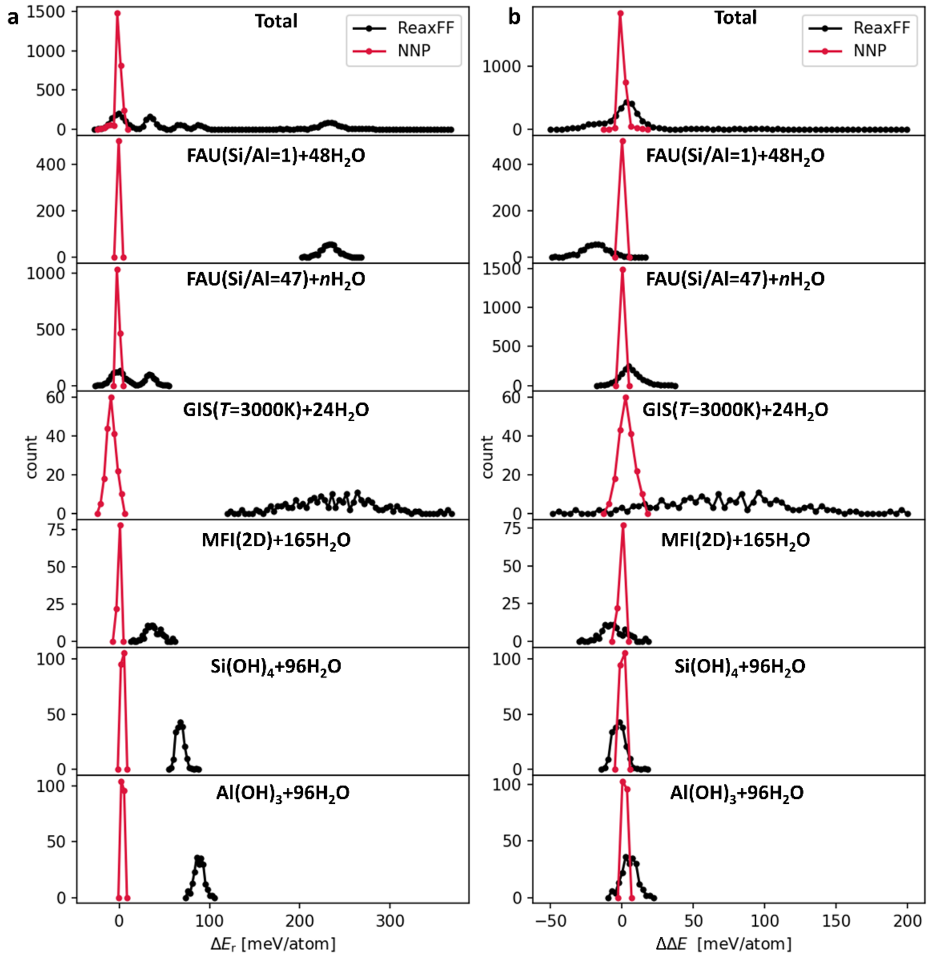

Supplementary Figure 4 Energy error distributions for all generalization tests of **a** the reaction energy E_r (see Eq 1) and **b** relative energies ΔE with respect to the initial structure of each MD trajectory (with same chemical composition).

Supplementary Table 3 NNP and ReaxFF RMSEs of reaction energies E_r (see eq 1), relative energies ΔE [meV atom^{-1}], and forces F [eV Å^{-1}] for all generalization tests (see Supplementary Figure 4b). The covariances (cov) are listed as well quantifying the correlation between the DFT total energies and forces (E_{DFT}, F_{DFT}) with the NNP errors (ΔE_{tot}, ΔF).

Generalization tests	NNP					Reax		
	ΔE_r	$\Delta\Delta E$	ΔF	cov(E_{DFT}, ΔE_{tot})	cov(F_{DFT}, ΔF)	ΔE_r	$\Delta\Delta E$	ΔF
FAU(Si/Al=1)+48H_2O	0.86	0.90	0.11	0.59	0.24	233.9	20.94	2.38
FAU(Si/Al=47)+1H_2O	3.08	0.5	0.06	0.16	0.10	8.61	11.34	1.60
FAU(Si/Al=47)+4H_2O	2.74	0.5	0.06	0.12	0.01	7.00	8.20	1.39
FAU(Si/Al=47)+48H_2O	0.78	0.7	0.09	0.30	0.14	34.81	7.64	1.13
GIS(T=3000K)+24H_2O	10.0	6.07	0.28	0.41	0.15	242.2	83.42	3.87
MFI(2D)+165H_2O	1.39	1.47	0.16	0.38	0.07	38.3	9.82	2.43
$Si(OH)_4$+96H_2O	4.13	1.32	0.12	0.29	0.18	67.6	4.94	0.59
$Al(OH)_3$+96H_2O	4.07	2.24	0.12	0.20	0.18	88.6	7.40	0.67
**Average**	**3.8**	**1.91**	**0.11**	**0.31**	**0.13**	**147.6**	**25.54**	**1.96**

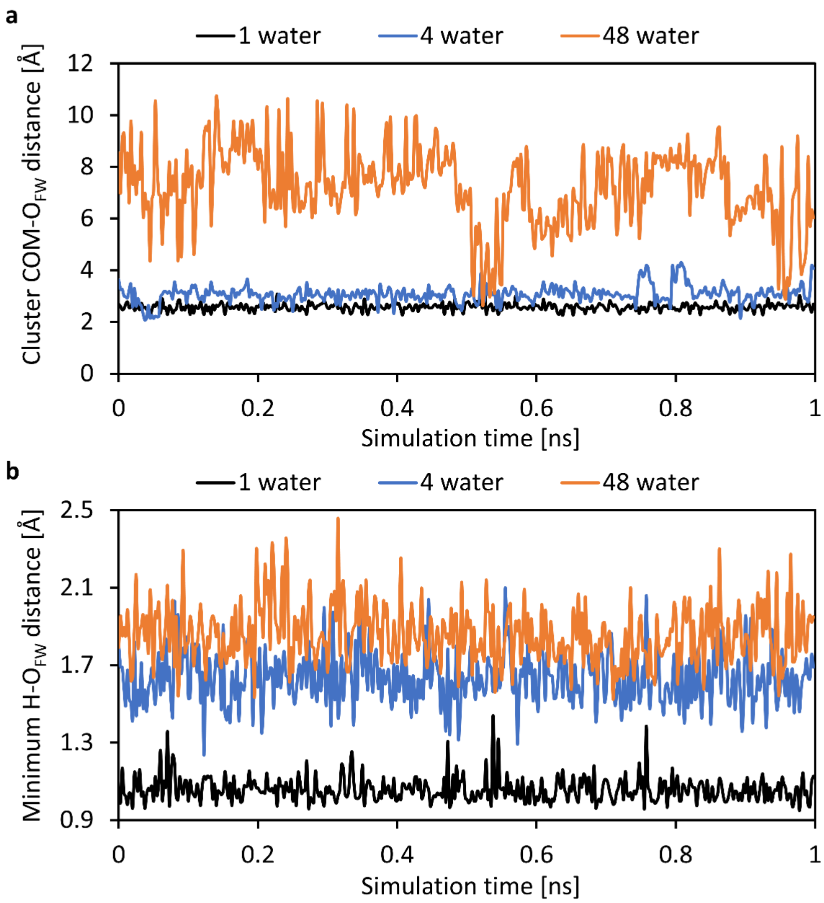

Supplementary Figure 5 Distance analysis of the (un)solvated proton in the FAU generalization test cases (Si/Al=47) with a single, four and 48 water molecules: **a** distance between the center-of-mass of the protonated cluster (Zundel or hydronium cation) to the closest lying Al-O_{FW}-Si framework oxygen, and **b** minimum distance between Al-O_{FW}-Si framework oxygens and all hydrogen atoms.

Supplementary Figure 5 shows the distance analysis of the (un)solvated proton and the center-of-mass (COM) of protonated water clusters for the OOD test cases FAU(Si/Al=47)+1H_2O, FAU(Si/Al=47)+4H_2O, and FAU(Si/Al=47)+48H_2O that contain only one Al site and proton. We considered the proton solvated if it is closer to a water oxygen than to a BAS (Al-O_{FW}-Si). A single water molecule adsorbs at the BAS leaving the proton mostly unsolvated with less than 3% solvated states of the 1 ns trajectory. The water tetramer (FAU(Si/Al=47)+4H_2O) solvates the proton throughout the 1 ns MD simulation but stays close to the BAS. In contrast, at full water loading (FAU(Si/Al=47)+48H_2O), protonated cluster (Zundel or hydronium cations) form and diffuse through the zeolite pore.

To test the impact of the NNP errors and energy/force covariances, we performed a "reverse" test simulation with a 10 ps AIMD run (SCAN+D3(BJ) level) using an optimized snapshot from the NNP level MD run for FAU(Si/Al=1)+48H_2O as initial structure. We chose this test case since the NNP trajectory showed hydronium-like species above the single six-rings (S6R) of the sodalite cages containing at least two aluminum atoms, that is not interacting with the bulk-like waters in the zeolite cage, which we denote as "pinned hydronium" (see Supplementary Figure 6a). This "pinned hydronium" was dynamically stable for the entire 10 ps AIMD run.

We recalculated the NNP energies and forces for the AIMD trajectory and Supplementary Figure 6b compares the NNP and SCAN+D3(BJ) calculated reaction energies E_r (see Eq 1) along with the NNP error ΔE_r. The NNP calculated E_r show an almost constant offset from the DFT values with an ΔE_r RMSE of 4.7 meV atom^{-1}. Similar to the test cases above, this offset is removed when using the relative energy error $\Delta\Delta E$ with an RMSE of 1.6 meV atom^{-1}. Both error metrics are somewhat larger compared to the NNP-driven MD run for FAU(Si/Al=1)+48H_2O but still lower than the NNP errors of the database test set (see Supplementary Table 2) and similar to other (rotationally invariant) state-of-the-art machine learning potentials.[7,9] This is probably connected to the NNP force errors (approx. 0.11 eV Å^{-1}) and larger covariances compared to other test cases which leads to sampling of a slightly different basin of the potential energy surface.

The transition from the NNP optimized structure to the SCAN+D3(BJ) basin can also be seen in Supplementary Figure 6c. It plots the NNP total energy error ΔE and force errors ΔF as a function of DFT calculated total energy and forces. In the initial equilibration stage of the AIMD run the system leaves the NNP level minimum towards the DFT level basin that is sampled for the rest of the AIMD simulation. The corresponding energy/force covariances (energy: 0.17, forces: 0.13) are lower than the analogous NNP-MD test case (FAU(Si/Al=1)+48H_2O).

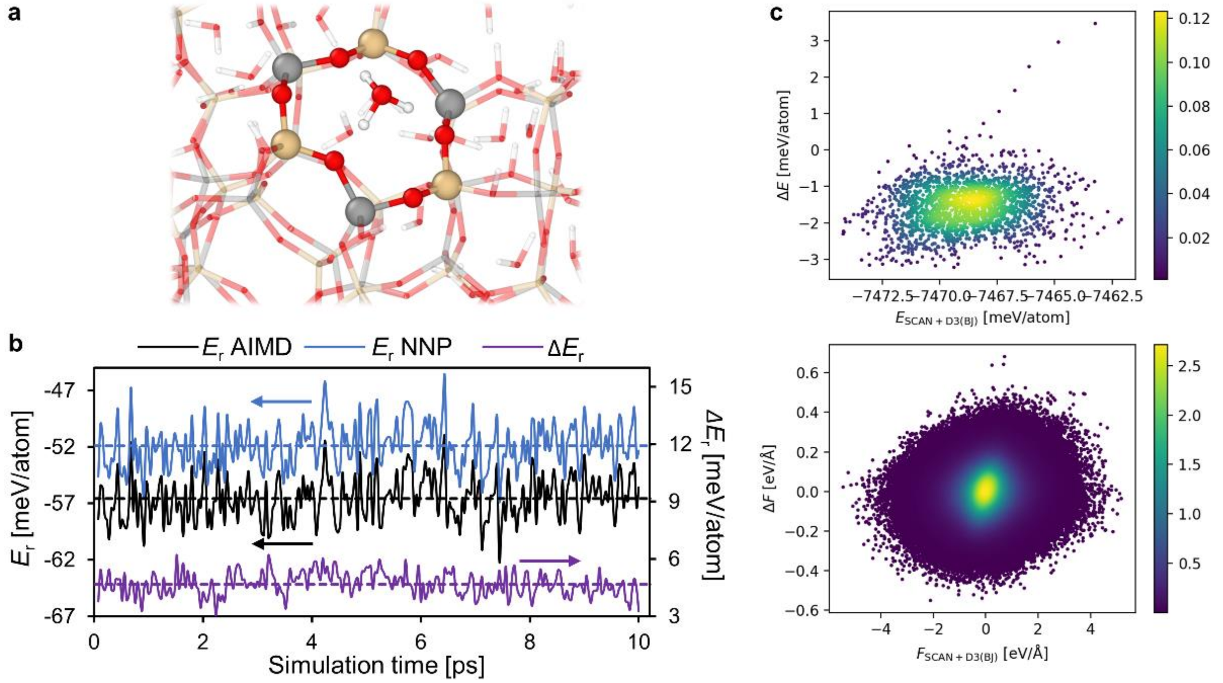

Supplementary Figure 6 AIMD (SCAN+D3(BJ) level) simulation of FAU(Si/Al=1)+48H_2O that shows a “pinned hydroxonium” species (**a**). We recalculated energy and forces at NNP level for the AIMD trajectory for NNP energy error evaluation (**b**). The NNP total energy error ΔE_{tot} and force errors ΔF as a function of DFT calculated total energy and forces are shown in **c** with covariances of 0.17 for energies and 0.13 for forces (color bars show the point density).

We performed another test on how the NNP energy/force errors change structure and energetics of DFT optimized zeolites by reoptimizing a test set of 7 purely siliceous structures (“Test (opt)” in Supplementary Figure 1) used in our previous work[1] on the development of NNPs for siliceous zeolites “NNP (silica only)”. Supplementary Table 4 lists the results of structure optimization at the DFT (PBE+D3(BJ), SCAN+D3(BJ)) and NNP level (NNP (this work), NNP (silica only)) for silica structures where experimental data (structures and enthalpies) are available. Both NNP versions have similar deviations from experiment as the used exchange-correlation functionals for bonding distances, atomic densities, and phase transition energies (enthalpies). In addition, the NNPs trained in this work have even better agreement with experiment than the NNPs (silica only).

In our previous work (Ref. 1), 1000 siliceous frameworks were randomly selected from the hypothetical (siliceous) zeolite database[1] for single-point calculations at the SCAN+D3(BJ) level as an OOD test set for the previously developed NNP (silica only). Here, we calculated energy and forces for this OOD test set using the NNPs (this work) to test if the new NNPs retain the capability to model hypothetical zeolites (“Test (SP)” in Supplementary Figure 1). Both NNPs, trained in this work and the silica only version, have approximately the same force RMSE of 73 and 76 meV Å^{-1}, respectively. The energy RMSE ΔE_r slightly increased from 3.8 meV atom^{-1} for NNP (silica only) to 7.2 meV atom^{-1} for NNP (this work) which is still about an order of magnitude lower than other approximations like ReaxFF or tight-binding DFT (RMSE about 100 meV atom^{-1}, see Ref. 1 for details).

Supplementary Table 4 Relative energies ΔE [kJ/mol Si], density ρ [Si/nm³], average Si-O bond distances d(Si-O) [Å] and mean average deviations (MAD) of lattice parameters ΔL [Å] from experiments calculated at the PBE+D3(BJ), SCAN+D3(BJ), NNP (silica only, Ref. 1) and NNP (this work) level for α-quartz (qu), α-cristobalite (cr), tridymite (tri) and purely siliceous zeolites. Experimental values for relative transition enthalpies ΔH and structural parameters were taken from Refs. 10–12. Values for SCAN+D3(BJ), PBE+D3(BJ) and NNP (silica only) were taken from Ref. 1.

	**EXP**			**SCAN+D3(BJ)**				**PBE+D3(BJ)**			
	**ΔH**	**ρ**	**d(Si-O)**	**ΔE**	**ρ**	**d(Si-O)**	**ΔL**	**ΔE**	**ρ**	**d(Si-O)**	**ΔL**
qu	0	26.52	1.61	0	26.61	1.61	0.01	0	25.92	1.62	0.04
cr	2.8	23.38	1.6	4.1	23.33	1.61	0	4	22.68	1.62	0.05
tr	3.2	22.35	1.56	6	22.16	1.6	0.1	6.5	21.61	1.62	0.13
AFI	7.2	16.89	1.61	10.5	17.16	1.6	0.06	12	16.89	1.62	0.04
FER	6.6	17.55	1.61	9.8	17.9	1.6	0.09	11.2	17.46	1.62	0.03
IFR	10	17.15	1.61	11	17.16	1.61	0.03	12	17.15	1.62	0.1
MTW	8.7	18.23	1.61	9.4	18.5	1.6	0.06	10.9	18.23	1.62	0.06
**MAD**	**-**	**-**	**-**	**2.1**	**0.18**	**0.01**	**0.05**	**3.0**	**0.3**	**0.02**	**0.06**
	**EXP**			**NNP (silica only)**				**NNP (this work)**			
	**ΔH**	**ρ**	**d(Si-O)**	**ΔE**	**ρ**	**d(Si-O)**	**ΔL**	**ΔE**	**ρ**	**d(Si-O)**	**ΔL**
qu	0	26.52	1.61	0	26.94	1.61	0.03	0.0	26.7	1.61	0.01
cr	2.8	23.38	1.6	6	22.14	1.61	0.1	5.3	22.9	1.61	0.03
tr	3.2	22.35	1.56	6.4	21.6	1.6	0.09	5.6	21.7	1.60	0.12
AFI	7.2	16.89	1.61	10.3	17.15	1.6	0.05	8.5	17.1	1.61	0.04
FER	6.6	17.55	1.61	10.8	17.85	1.6	0.08	9.1	17.8	1.60	0.07
IFR	10	17.15	1.61	11.3	16.86	1.61	0.06	9.8	17.0	1.61	0.04
MTW	8.7	18.23	1.61	10	18.28	1.6	0.02	8.0	18.5	1.61	0.08
**MAD**	**-**	**-**	**-**	**2.7**	**0.47**	**0.01**	**0.06**	**1.4**	**0.3**	**0.01**	**0.06**

Next, we evaluated the accuracy of NNP structure optimizations for the OOD aluminosilicate zeolite MFI(Si/Al=95) by calculating single water adsorption energies on all twelve (T1-T12) symmetry inequivalent T-sites. Supplementary Figure 7 depicts the adsorption energies for the twelve selected (BAS) T-sites at the NNP, SCAN+D3(BJ) and PBE+D3(BJ) level. The NNP water adsorption energies range between -80 and -110 kJ/mol. The mean absolute deviation (MAD) between the NNPs and their reference level SCAN+D3(BJ) is about 10 kJ mol^{-1}. For comparison, PBE+D3(BJ) has an MAD of 12 kJ mol^{-1} with respect to SCAN+D3(BJ) which means the NNPs provide approximately the same quality as another exchange-correlation functional.

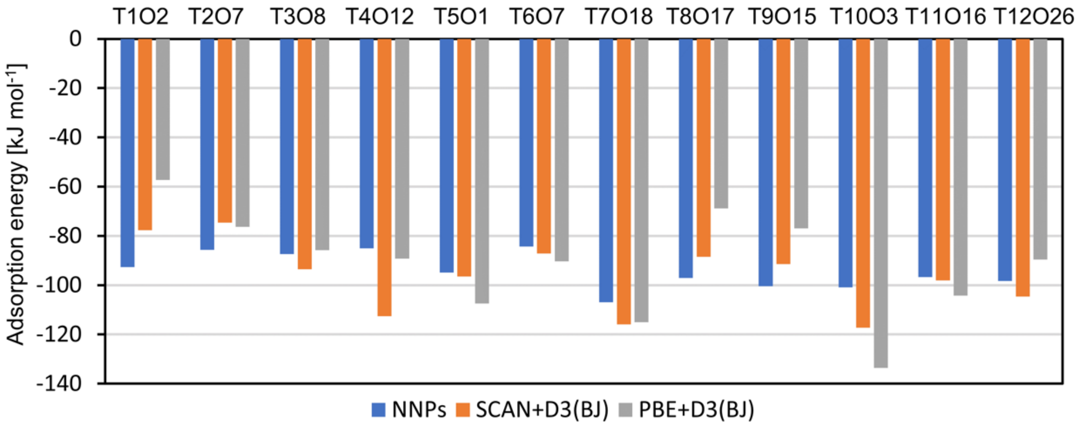

Supplementary Figure 7 Adsorption energies at the NNP, SCAN+D3(BJ), and PBE+D3(BJ) level for 12 different T-sites (BAS) in MFI (Si/Al = 95).

Supplementary Figure 8 depicts snapshots and relative energies Δ*E* calculated at the NNP and SCAN+D3(BJ) level for the generalization test case of liquid Gismondite GIS (Si/Al=1, 24 water, *T*=3000 K). The trajectory shows numerous reactive events including Si-O and Al-O bond breakages, silanol and aluminol formations under extremely high temperatures. The relative energy errors ΔΔ*E* of 6 meV atom^{-1} (see Supplementary Table 3) are small compared to the energy fluctuations in the 3000 K MD run of about 160 meV atom^{-1}.

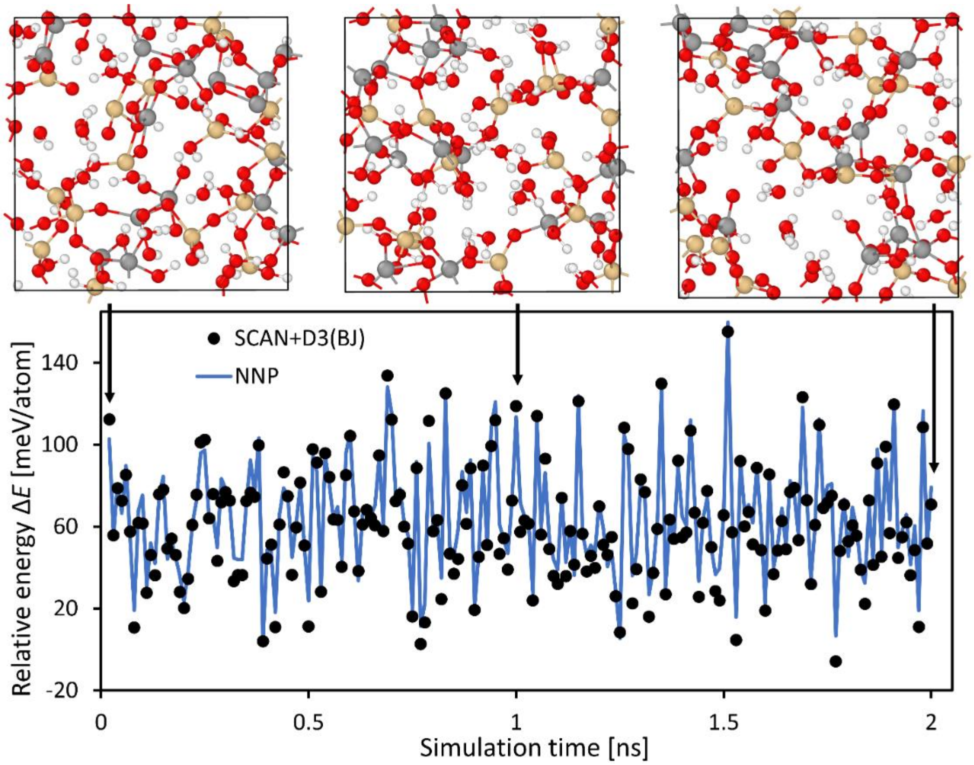

Supplementary Figure 8 MD trajectory snapshots of liquid GIS (Si/Al=1, 24 water, *T*=3000 K) and relative energies Δ*E* calculated at the NNP and SCAN+D3(BJ) level.

Supplementary Figure 9 depicts the reaction pathways and energy profiles of NEB calculations for an Si-O bond cleavage mechanism and a water assisted proton jump in FAU (OOD test case) calculated and the NNP and SCAN+D3(BJ) level. Supplementary Table 5 lists the corresponding relative energies Δ*E*.

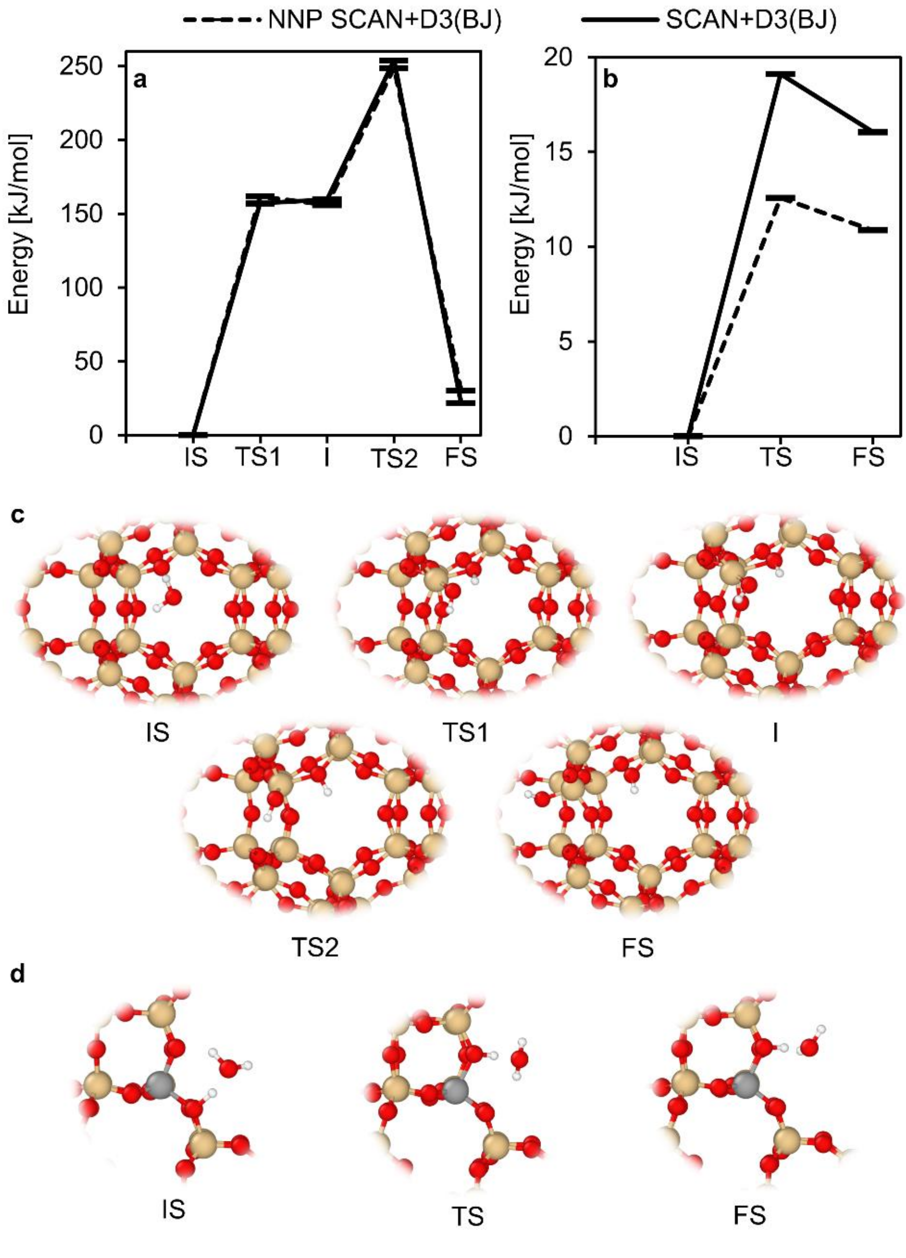

Supplementary Figure 9 Reaction paths of an Si-O(H) bond dissociation (**a**, **c**) and a water-assisted proton jump (**b**, **d**) for the OOD test case FAU. **a**, **b** Static NNP simulations and corresponding SCAN+D3(BJ) energies. **c**, **d** atomic structures along the reaction path (Si: yellow, Al: grey, O: red, H: white).

Supplementary Table 5 Relative energies Δ*E* (NNP and DFT level) of initial (IS), transition (TS) and final states (FS) for the water-assisted proton jump and Si-O(H) bond breakage shown in Supplementary Figure 9.

	Δ*E* [kJ/mol] NNP	SCAN+D3(BJ)
**Proton jump + H_2O**		
IS	0.0	0.0
TS	12.6	19.1
FS	10.9	16.1
**Si-O(H) bond breakage**		
IS	0	0
TS1	161.8	157.1
I	156.0	159.7
TS2	248.9	253.9
FS	30.2	21.9

Supplementary Figure 10 shows the test set energy and force errors of the ΔNNP model as a function of training set size. The training dataset contained ωB97X-D3(BJ) calculated energies and forces for 500 structures taken from a biased dynamics run for the proton jump in CHA (see Figure 3). Taking more than 150 configurations for the ΔNNP training set gives only negligible accuracy improvements in energy and forces.

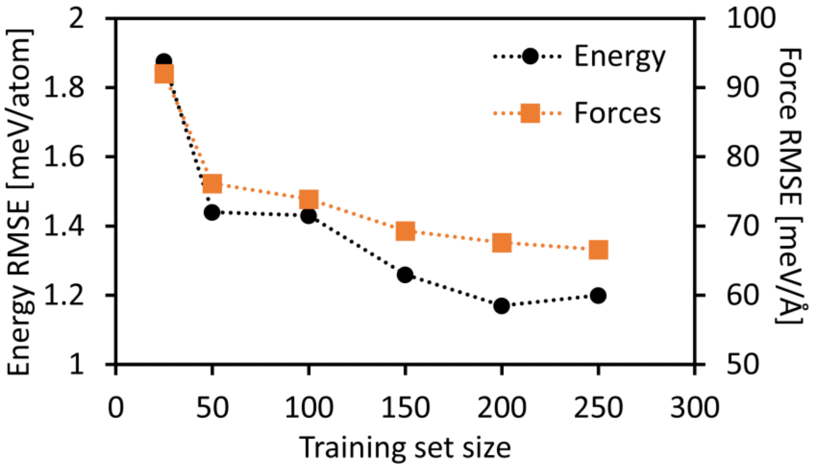

Supplementary Figure 10 Test set errors of ΔNNP model as a function of training set size. The training dataset contains ωB97X-D3(BJ) calculated energies and forces of structures taken from a biased dynamics run of the proton jump in CHA (see Figure 3).

Supplementary Table 6 lists the relative energies Δ*E* and reaction energies E_r (ΔNNP and ωB97X-D3(BJ) level) for the Al-O(H) bond breakage and proton jump mechanism in FAU which is a generalization (OOD) test case for the ΔNNP model. The ΔNNP reaction energies E_r show an almost constant offset of about 2.5 eV from their ωB97X-D3(BJ) reference level. However, similar to the NNP generalization tests (see Supplementary Table 3), the relative energies deviate much less from their reference DFT level, here by less than 8.7 kJ mol^{-1} with an MAD of 5.6 kJ mol^{-1} for the tested OOD reaction pathways.

Supplementary Table 6 Relative energies Δ*E* and reaction energies E_r (ΔNNP and DFT level) of initial, transition and final states obtained from ΔNNP level NEB calculations for the reactions in FAU shown in Figure 3 (OOD test cases for the ΔNNP model).

	E_r [eV]		Δ*E* [kJ/mol]	
	**ΔNNP**	**ωB97X-D3(BJ)**	**ΔNNP**	**ωB97X-D3(BJ)**
**Al-O(H) bond breakage**				
IS	3.656	6.172	0.0	0.0
TS1	4.626	7.052	93.2	84.5
I	4.366	6.802	68.4	60.3
TS2	4.416	6.882	73.6	68.4
FS	4.156	6.662	48.4	46.7
**Proton jump**				
IS	4.703	6.705	0.0	0.0
TS	5.433	7.485	71.0	75.6
FS	4.773	6.725	7.3	2.2

To further validate the ΔNNP accuracy and generalization capability, we subsampled 200 structures from the Metadynamics runs for the Al-O(H) bond breakage and proton jump mechanism in FAU shown in Figure 3 (OOD test cases for the ΔNNP model) and performed SP calculations at the ωB97X-D3(BJ) level. Supplementary Table 7 lists the reaction energy errors ΔE_r (see Eq 1), relative energy errors ΔΔ*E* and force errors Δ*F* of the ΔNNP model with respect to its reference ωB97X-D3(BJ) level. Supplementary Figure 11 shows the corresponding energy error distributions. Again, similar to the above mentioned OOD tests, the ΔE_r distributions show an almost constant offset to their DFT reference leading to ΔE_r RSMEs of 15 to 18 meV atom^{-1}. However, the relative energy errors ΔΔ*E* of less than 2 meV atom^{-1} and Δ*F* of less than 100 meV Å^{-1} show that the ΔNNP model is capable of modeling different reactions in an OOD zeolite framework with similar chemical composition. This means that the ΔNNP model, even if trained only on a proton jump in CHA, is robust enough to describe relative positioning of the stationary states for an OOD system of interest. However, it fails to generalize across (in between) different systems and chemical composition, e.g., it would fail if used for cohesion energies or comparison across zeolite frameworks.

Supplementary Table 7 ΔNNP errors of reaction energies ΔE_r and relative energies ΔΔ*E* [meV atom^{-1}] and force errors Δ*F* [meV Å^{-1}] for a subset of 200 structures from Metadynamics runs of the proton jump and Al-O(H) bond breakage for the OOD test case FAU (see Figure 3).

	Proton jump			Al-O(H) bond breakage		
	ΔE_r	ΔΔ*E*	Δ*F*	ΔE_r	ΔΔ*E*	Δ*F*
MAE	14.90	0.68	77.91	18.04	1.81	76.97
RMSE	14.92	0.80	98.26	18.06	1.95	98.76

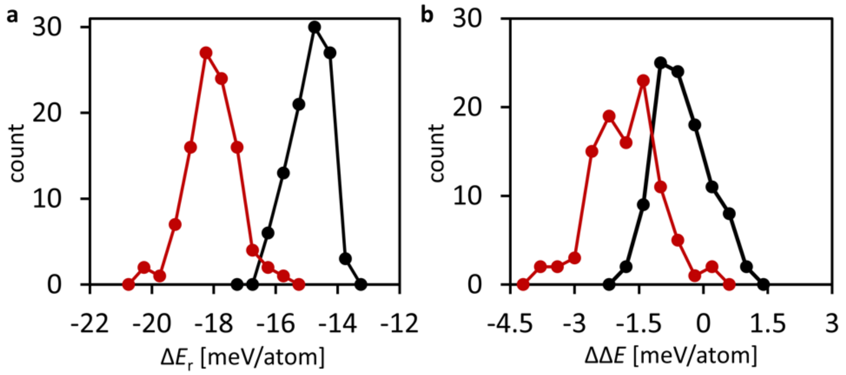

Supplementary Figure 11 Energy error distributions of the ΔNNP model for biased dynamics runs of the proton jump (black) and Al-O(H) bond breakage (red) in FAU (see Supplementary Table 6): **a** reaction energy E_r (see Eq 1) and **b** relative energies Δ*E* errors.

Supplementary Figure 12 depicts the free energy profiles of the proton in CHA (see Figure 3) as test case that is within the NNP training domain (ID test case) calculated at the NNP and SCAN+D3(BJ) level. The simulations were run at 300 K, with the time step of 0.5 fs, height of Gaussians set to 2.7 kJ mol^{-1}, and with the hydrogen replaced by tritium. In addition, we tested two different Metadynamics setups at the NNP level: i) deposition rate set to 200 (i.e., every 100 fs) and the Gaussian width to 0.04 Å (MTD1), and ii) deposition rate set to 50 (i.e., every 25 fs) and the Gaussian width to 0.02 Å (MTD2). The deviations due to MTD setup are about 10 kJ mol^{-1}, which is about the same as deviation between DFT-reference and the NNP.

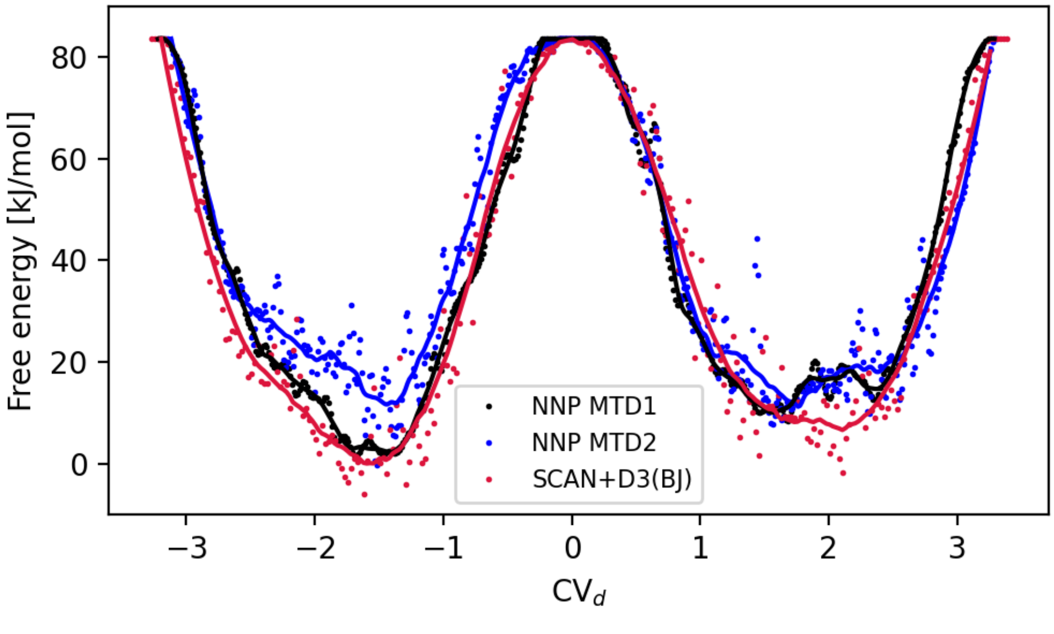

Supplementary Figure 12 Calculated free energy profiles using AIMD (SCAN+D3(BJ) level) and NNP level simulations with two different setups (MTD1 and MTD2) for the proton jump in CHA (see Figure 3).

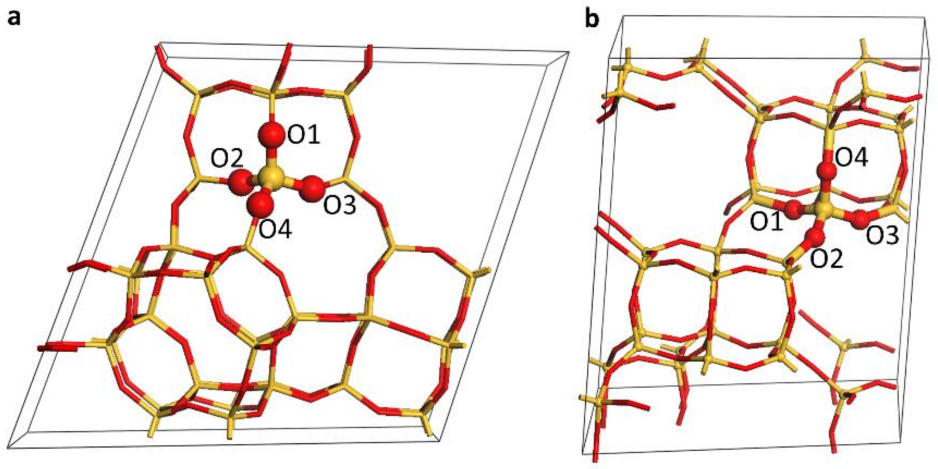

Supplementary Figure 13 Numbering of symmetry inequivalent oxygen atoms (as labeled in the IZA database) for **a** the primitive unit cell of FAU and **b** CHA.

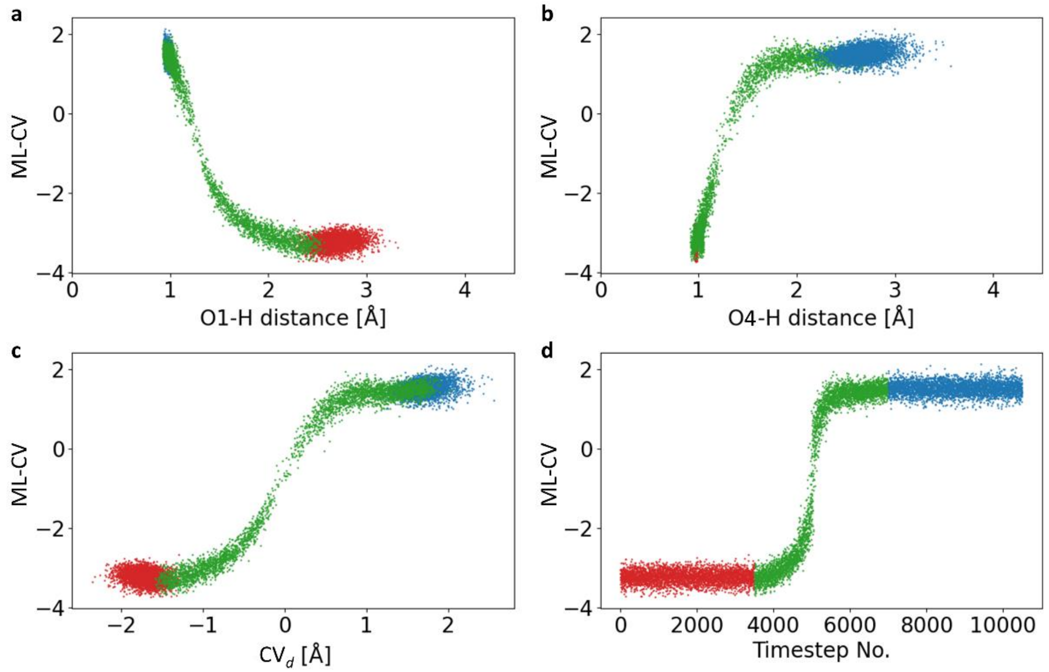

Supplementary Figure 14 Projections of machine learned collective variables (ML-CV) to the distances between the proton and oxygens O1 and O4 (**a**, **b**) for the proton jump in FAU (see Figure 3). The ML-CVs were trained on equilibrium MD runs of reactants (red points) and products (blue points). Steered dynamics runs (green points) using the expert-chosen CV_d (difference of O1-H and O4-H distance) were used for verification (**c**) but not for training of the ML-CV. **d** Evolution of the ML-CV for the simulated data points (concatenated from all MD runs keeping the order of simulation steps). The ML-CV smoothly interpolates between reactant and product state similar to the chosen ("standard") CV_d.

Supplementary Table 8 Parameters for well-tempered metadynamics runs. We refer to the PLUMED software documentation of the function *METAD* for the meaning of the keywords.

Reaction	Pace	Biasfactor [kJ mol^{-1}]	T [K]	Height [kJ mol^{-1}]	σ [Å]	Grid [Å]	Grid bin
Proton jump	200	35	300	2.8	0.02	[-1.5, 1.5]	1000
Al–O(H) bond breakage	200	35	300	2.1	0.02	[-1.5, 1.5]	1000

3. Supplementary references

1. Erlebach, A., Nachtigall, P. & Grajciar, L. Accurate large-scale simulations of siliceous zeolites by neural network potentials. *npj Comput. Mater.* **8**, 1–12 (2022).
2. Crum, J. T., Crum, J. R., Taylor, C. & Schneider, W. F. Characterization and analysis of ring topology of zeolite frameworks. *Micropor. Mesopor. Mat.* **351**, 112466 (2023).
3. Eldar, Y., Lindenbaum, M., Porat, M. & Zeevi, Y. Y. The farthest point strategy for progressive image sampling. *IEEE Trans. Image Process* **6**, 1305–1315 (1997).
4. Imbalzano, G. *et al.* Automatic selection of atomic fingerprints and reference configurations for machine-learning potentials. *J. Chem. Phys.* **148**, 241730 (2018).
5. Bartók, A. P., Kondor, R. & Csányi, G. On representing chemical environments. *Phys. Rev. B* **87**, 184115 (2013).
6. Schütt, K., Unke, O. & Gastegger, M. Equivariant message passing for the prediction of tensorial properties and molecular spectra. in *Proceedings of the 38th International Conference on Machine Learning* 9377–9388 (PMLR, 2021).
7. Bocus, M. *et al.* Nuclear quantum effects on zeolite proton hopping kinetics explored with machine learning potentials and path integral molecular dynamics. *Nat. Commun.* **14**, 1008 (2023).
8. Schütt, K. T., Sauceda, H. E., Kindermans, P.-J., Tkatchenko, A. & Müller, K.-R. SchNet - A deep learning architecture for molecules and materials. *J. Chem. Phys.* **148**, 241722 (2018).

9. George, J., Hautier, G., Bartók, A. P., Csányi, G. & Deringer, V. L. Combining phonon accuracy with high transferability in Gaussian approximation potential models. *J. Chem. Phys.* **153**, 044104 (2020).

10. Piccione, P. M. *et al.* Thermochemistry of Pure-Silica Zeolites. *J. Phys. Chem. B* **104**, 10001–10011 (2000).

11. Baerlocher, Ch., Meier, W. M. & Olson, D. M. *Attas of Zeolite Framework Types*. (Elsevier, Amsterdam, 2001).

12. Baerlocher, Ch. & McCusker, L. B. Database of Zeolite Structures: http://www.iza-structure.org/databases/ (accessed: February 8, 2023).
